# Single-site robotic cholecystectomy: comparison of clinical outcome and the learning curves in relation to surgeon experience in a community teaching hospital

**DOI:** 10.1186/s12893-018-0373-8

**Published:** 2018-06-11

**Authors:** Mohamad Dughayli, Sergey Shimunov, Sherry Johnson, Fadi Baidoun

**Affiliations:** Department of General Surgery, Henry Ford Wyandotte Hospital, 2333 Biddle Avenue, Wyandotte, MI 48192 USA

**Keywords:** Single-site, Robotic surgery, Cholecystectomy

## Abstract

**Background:**

The aim of this study was to analyze the experience of two surgeons who have different laparoscopic skills and case volume, with single-site robotic cholecystectomy (SSRC) and evaluate their learning curves.

**Methods:**

All SSRCs performed between March 2013 and April 2015 were retrospectively reviewed. The patients were divided among two surgeons based on the surgeon’s level of laparoscopic skills and case volume. Surgeon 1 had experience in advanced laparoscopy while surgeon 2 had basic laparoscopic skills. Patients’ demographic data, intraoperative and short-term postoperative results were assessed.

**Results:**

The analysis included 102 patients for surgeon 1 and 15 patients for surgeon 2. There were no major or minor complications in either cohort. Operative time was significantly longer for surgeon 2, conversion to laparoscopy rate was 4% for surgeon 1 compared to 20% for surgeon 2, *P* = 0.044, which is statistically significant.

**Conclusions:**

SSRC is an easy and safe procedure that can be quickly learned and performed in a reproducible manner by surgeons with experienced laparoscopic skills and greater case volume.

## Background

Laparoscopic cholecystectomy is the most common laparoscopic procedure and is the gold standard for treatment of gallbladder disease. Single-incision laparoscopic cholecystectomies aimed to reduce the number of incisions, and therefore, reduce the risk of surgical site infection, scarring and postoperative pain while allowing earlier return to the patient’s activities of daily living. Navarra et al. in 1997 [1] performed the first single-incision laparoscopic cholecystectomy via the trans-umbilical approach. Although promising results have been reported [2] in terms of safety and technique feasibility, others have reported increased complications, bile duct injuries [3–5] and incisional hernia occurrence. The single-incision port approach had significant limitations, mainly associated with instrumentation and proper triangulation [6], image instability, instrument collision, limited range of motion and decrease in the amount of force available at the tips of flexible instruments.

Robotic surgery has become an established alternative to conventional laparoscopy by solving the problems of angulation, improving the ergonomics of single-incision technologies [7], and overcoming the intrinsic limitations of single-incision laparoscopy, as reported by Kroh et al. [8].

Henry Ford Wyandotte Hospital, a community based teaching hospital, initiated its robotic surgery program in March 2013. Our team performed the first operation using the single-site platform for single-incision surgery with the da Vinci Si system (Intuitive Surgical, Sunnyvale, CA). Plans were made to expand the application of this new technology in our teaching hospital. As with most operative procedures, case volume and the surgeon’s expertise are considered to have a major influence on procedure success, learning curve as measured by the total operative time and complication rates. In our hospital, surgeries were performed by two surgeons with different levels of laparoscopic skills and overall surgical experience and case volume. The primary aim of this study was to evaluate whether intraoperative and postoperative measures differ significantly based on a surgeon’s previous laparoscopic experience and case volume, with the focus on factors such as operative time as a reflection of the learning curve, conversion to laparoscopy, hospital stay, and previous abdominal surgery. Moreover, the learning curve was noted to be greatly influenced by the surgeon’s experience and patient prior surgeries. In this report we assess the learning curve based on two surgeons’ experience and case volume.

## Methods

This retrospective study was approved by the Henry Ford Health System Institutional Review Board (No. 10555). There were 117 patients who underwent single-site robotic cholecystectomy (SSRC) between March 2013 and April 2015 at our institution. Variables including demographics, operative time, estimated blood loss, length of stay, previous abdominal surgeries, postoperative hemorrhage and operative times among patients with previous abdominal surgeries, a variable generally excluded in previously published SSRC studies, were compared between two surgeons based on the surgeon’s level of experience. Surgeon 1 had training in advanced laparoscopy, greater case volume and more than 10 years of experience and surgeon 2 had basic laparoscopic skills, less case volume and was at the beginning of his first 2 years of practice.

The indications of the procedure were chronic cholecystitis, biliary colic, biliary dyskinesia, and gallstone pancreatitis in both inpatient and outpatient settings. Acute cholecystitis diagnosed pre-operatively served as the only exclusion criterion. There were no exclusions based on body mass index or previous abdominal surgery.

Patients were assigned a case identification number sequentially by date of surgery (i.e., Case #1 corresponds with the earliest date in the dataset [March 11, 2013] and Case #117 corresponds to the last date in the dataset [June 1, 2015]). Patient demographic data and information regarding patient comorbidities, indications for surgery, complications, rate of conversion, average length of hospital stay, and total operative time were collected. Operative time was recorded electronically and was measured from the beginning of skin incision until subcuticular skin closure. Data compared for both groups of surgeons included age, sex, body mass index, diagnosis, conversion rate, intraoperative and postoperative complications, and postoperative outcome.

After surgery all patients are seen in the office at 2 weeks, then at 6 weeks for evaluation. Meanwhile patients were counseled regarding signs and symptoms of complications, including developing hernias, and were asked to notify their surgeons regarding any concerns. To assess the learning curve, the operative times were compared between the two surgeons.

### Technique

A 2.5-cm vertical incision is made through the umbilicus, carried down to the fascia, and the peritoneum to accommodate the port [9]. A single-site silicon port was used with an 8.5-mm high-definition endoscope. After establishment of the pneumoperitoneum and docking the robot, the camera and two curved single-site cannulas were inserted as well as a 5-mm trocar for the assistant. The robotic surgical system automatically establishes an association between the hands of the surgeon and the ipsilateral tip of the surgical instrument enabling intuitive control.

After exploring the abdominal cavity, the assistant grasped the fundus of the gallbladder to expose the sub-hepatic space. At the console, the surgeon performs the dissection of the gallbladder in a similar fashion to laparoscopic cases. Both the cystic duct and the cystic artery are dissected with the monopolar hook or Maryland dissector and divided between clips (Hem-O-Lock medium-large clips, Teleflex Incorporated, Morrisville, NC). Using electrocautery, further dissection of the gallbladder is continued until its detachment from the liver bed. The single-site port is removed through the umbilical incision along with the specimen, after undocking the robot and removing the instruments and camera. The fascial defect is closed with a running 0-Vicryl (Ethicon Inc., Somerville, NJ) suture and the skin with a 4.0-Vicryl subcuticular continuous suture.

### Statistical analysis

All continuous variables are described using means and standard deviations, while all categorical variables are described using counts and percentages. Two-group comparisons are carried out using Wilcoxon rank-sum tests for continuous variables due to the small group sizes and non-Gaussian distributions and using chi-square or Fisher’s exact tests as appropriate for categorical variables. Statistical significance is set at *P* < 0.05. All analyses are performed using SAS 9.4 (SAS Institute Inc., Cary, NC).

## Results

Between March 2013 and April 2015, 117 SSRC cases were performed by two surgeons based on their laparoscopic skills and experience (Table 1). Surgeon 1 had a larger number of patients (*n* = 102) compared to surgeon 2, who had 15. There were 24 males (24%) and 78 females (76%) in the series of patients for surgeon 1 compared to surgeon 2 who had 5 males (33%) and 10 females (67%). No statistically significant differences were found for patient age mean ± standard deviation 44.6 ± 16.6 years and 43 ± 20.8 years for groups 1 and 2, respectively, *P* = 0.572), body mass index 28.8 ± 5.6 and 27.9 ± 8.6 for surgeons 1 and 2, respectively, *P* = 0.185) or for American Society of Anesthesiologists 1–4 classification using univariate analysis (*P* = 0.548). No other significant differences were found except for preoperative diagnosis of biliary colic, which was 2% for surgeon 2 compared to 0% for surgeon 1 (*P* = 0.24).

**Table 1:** Descriptive statistics and univariate surgeon comparisons

Variable	Response	All (*N* = 117)	Surgeon 1 (*N* = 102)	Surgeon 2 (*N* = 15)	P*
Age	N, Mean ± SD	117, 44.4 ± 17.1	102, 44.6 ± 16.6	15, 43.0 ± 20.8	0.572
Sex	Male	29 (25%)	24 (24%)	5 (33%)	0.522
	Female	88 (75%)	78 (76%)	10 (67%)	
BMI (kg/m2)	N, Mean ± SD	117, 28.7 ± 6.1	102, 28.8 ± 5.6	15, 27.9 ± 8.6	0.185
ASA	N, Mean ± SD	117, 2.0 ± 0.6	102, 2.0 ± 0.5	15, 2.1 ± 0.7	0.548
Preoperative diagnosis	Biliary colic	2 (2%)	0 (0%)	2 (2%)	**0.024**
	Biliary dyskinesia	1 (1%)	1 (7%)	0 (0%)	
	Chronic cholecystitis	41 (35%)	2 (13%)	39 (38%)	
	Gallstone pancreatitis	4 (3%)	0 (0%)	4 (4%)	
	Symptomatic cholelithiasis	69 (59%)	12 (80%)	57 (56%)	

*ASA* American Society of Anesthesiologists, *BMI* body mass index

*Statistically significant differences between the 2 surgeons are noted with a bold *p*-value

Table 2 contains intraoperative and postoperative parameters and all patients were stratified by surgeon experience. Forty-three of surgeon 1’s patients (42%) had previous abdominal surgery compared to 2 (13%) of surgeon 2 (*P* = 0.045). Intraoperative cholangiography was not required in any of the cases. Additional ports were required in 6 patients, 3 for surgeon 1 (3%) versus 3 for surgeon 2 (20%, *P* = 0.027). Four of the patients of surgeon 1 (4%) were converted to multiport laparoscopic cholecystectomy compared to 3 (20%) for surgeon 2 (*P* = 0.044). One of surgeon 2’s conversions was attributed to the cannulas being too long, which made it technically not safe to proceed with the dissection. Postoperative outcomes were similar between the 2 surgeons; no one had a postoperative hemorrhage or bile leak. The average length of hospital stay in surgeon 1’s patients was 1.5 ± 1.3 days as compared to 1 ± 0.0 days for surgeon 2’s patients; the variation is due to the that fact some of the patients were admitted to the hospital for other medical conditions.

**Table 2:** Intraoperative and postoperative parameters - univariate surgeon comparisons

Variable	Response	All (*N* = 117)	Surgeon 1 (*N* = 102)	Surgeon 2 (*N* = 15)	P*
Operative time (min)	N, Mean ± SD	117, 49.3 ± 17.8	102, 45.0 ± 12.7	15, 78.8 ± 19.5	**< 0.001**
Single incision		109 (93%)	97 (95%)	12 (80%)	0.065
Additional port		6 (5%)	3 (3%)	3 (20%)	**0.027**
Converted to laparotomy		7 (6%)	4 (4%)	3 (20%)	**0.044**
IOC		0	0	0	N/A
Incision length (cm)	1.5	1 (1%)	0 (0%)	1 (25%)	**< 0.001**
	2	2 (2%)	0 (0%)	2 (50%)	
	2.5	103 (97%)	102 (100%)	1 (25%)	
EBL	N, Mean ± SD	117, 6.6 ± 9.7	102, 6.8 ± 10.3	15, 5.0 ± 0.0	> 0.999
Hospital stay		24 (21%)	21 (21%)	3 (20%)	> 0.999
Days post-OR	N, Mean ± SD	25, 1.5 ± 1.2	22, 1.5 ± 1.3	3, 1.0 ± 0.0	0.466
Postoperative hemorrhage		0	0	0	N/A
Previous abdominal surgery		45 (38%)	43 (42%)	2 (13%)	**0.045**
Days between surgeries	N, Mean ± SD	116, 7.0 ± 19.5	101, 5.2 ± 10.9	15, 19.0 ± 46.0	0.910

*EBL* estimated blood loss, *IOC* intraoperative cholangiography, *OR* operating room, *SD* standard deviation

*Statistically significant differences between the 2 surgeons are noted with a bold *p*-value

**Fig. 1:**
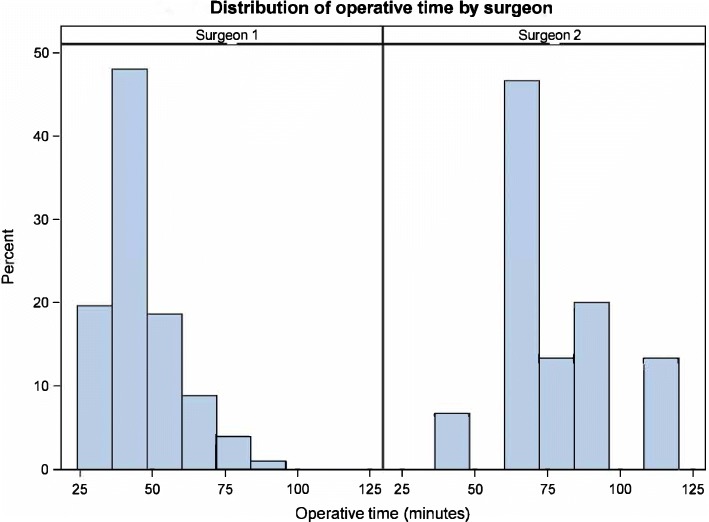
Distribution of operative times by surgeons 1 and 2

**Fig. 2:**
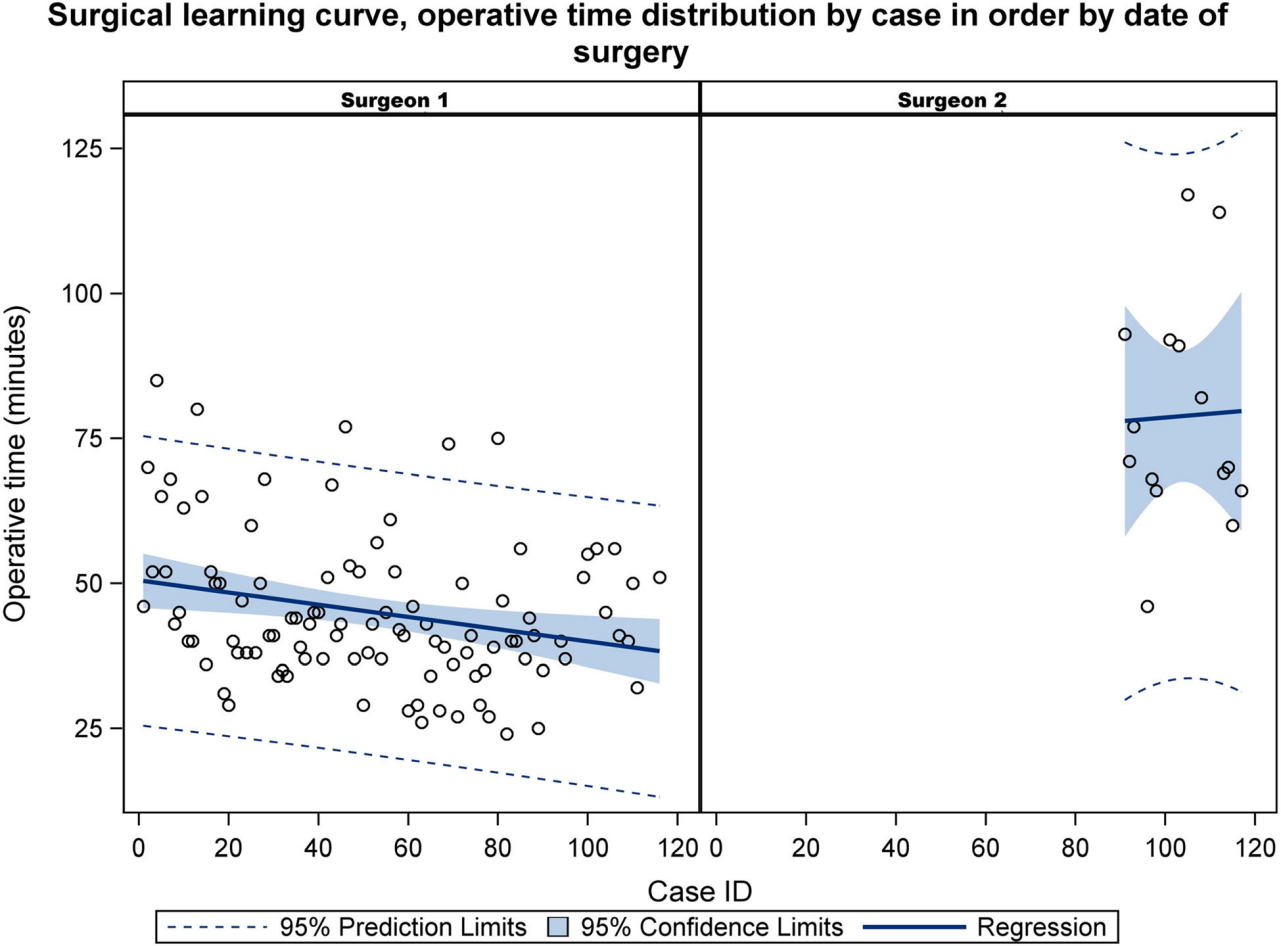
Surgical learning curve, operative time distribution by case in order by date of surgery

Figures 1 and 2 Surgeon 1 had a significantly lower average operative time (45.0 ± 12.7 min) compared to surgeon 2 (78.8 ± 19.5 min, *P* < 0.001) (Table 1 and Figs. 1 and 2). A trend of decreasing operative time that fit a line of regression was observed in the cases performed by surgeon 1, starting at case #40, extending through case #102 (Fig. 2). It was noted that some variations exist with scattered blots above the line due to patients having previous abdominal surgeries (Fig. 2). Unfortunately a learning curve could not be inferred for surgeon 2 due to the smaller number of cases.

Patients with previous abdominal surgeries was another variable found to be significantly higher among surgeon 1’s patients (42%) compared to surgeon 2’s patients (13%) (*P* = 0.045). On the other hand, when the operative time was stratified by whether or not the patients had a previous abdominal surgery (Table 3), it was shown that the operative time in 59 patients with no previous abdominal surgery was 45.6 ± 12.9 min for surgeon 1 compared to 73.2 ± 13.6 min in 13 patients of surgeon 2, *P* < 0.001. In the series of 43 patients of surgeon 1 who had previous abdominal surgeries, the mean operative time was 44.0 ± 12.6 min as compared to 115.5 ± 2.1 min for surgeon 2’s patients with previous abdominal surgeries, *P* = 0.019. In both instances, patients with and without previous abdominal surgeries, the mean operative time is significantly higher for surgeon 2 as compared to surgeon 1. For case volume, the average number of cases performed on a single day was 1.3/day for surgeon 1 and 1.1/day for surgeon 2. The average number of days between procedures is 6.5 days for surgeon 1 vs 38.8 days for surgeon 2. There was no significant difference in patient-related factors such as age and sex. Additionally, the two series of patients for both surgeons did not significantly differ in any of the following: average length of hospital stay (*P* > 0.999), intraoperative complications, postoperative complications, readmissions, or reoperations. None of the patients received a cholangiogram.

**Table 3:** Operative time by surgeon, stratified by surgery status

Variable	Response	Surgeon 1	Surgeon 2	*P*
Operative time, no previous abdominal surgery	N, Mean ± SD	59, 45.6 ± 12.9	13, 73.2 ± 13.6	< 0.001
Operative time, previous abdominal surgery	N, Mean ± SD	43, 44.0 ± 12.6	2, 115.5 ± 2.1	0.019

*SD* standard deviation

## Discussion

This study investigated the impact of surgeon experience on the perioperative and postoperative functional outcome of SSRC utilizing a single-site robotic platform technology. The total operative time, a reflection of the learning curve among two surgeons with different laparoscopic skills and case volume, was assessed and compared to the existing literature.

The introduction of the robotic single-site platform in 2011 has resolved the limitations of single-incision laparoscopy. The procedure has become easy to master with robotic assistance with functional results similar to those of conventional laparoscopy [10]. The curved trocars were designed to diminish the triangulation problem and four access cannulas in the trocar eliminate the need for percutaneous suture retraction [11]. The three-dimensional high-definition view provides the additional advantage of better visualization of the Calot’s triangle, so the dissection phase of the cystic artery and duct can be performed more safely. However, the da Vinci Si robotic system has its own limitations; it has a considerable size that limits the space in the operating room for the circulating staff while the arms of the robot limits the access of the bedside assistant [11].

According to Pietrabissa et al. [12], “In surgery an important factor in deciding whether to embrace a new technology for an old problem is the degree of difficulty in mastering the new skill set.” Completing the task in a timely manner is the parameter most commonly used to objectively evaluate a learning curve. Four comparative studies on conventional four-port laparoscopic versus multiport robotic cholecystectomy have described reaching equivalent operative times using the robot after a learning curve of 20 to 50 procedures [13–16]. Most reported studies observed a learning curve to be an important component of the total operative time [13, 14]. Although experienced laparoscopic surgeons can safely perform a single-site laparoscopic cholecystectomy in about the same time they do using the four-port technique [15], the steep learning curve and potential complications of this technology [17] might have prevented its implementation on a larger scale. Solomon and colleagues [18] reported a learning curve of approximately 10 cases to master the single-incision laparoscopic cholecystectomy, dropping the average operative time from 110 min to 75 min.

Single-site robotic platform technology provided the surgeon more flexibility, and better visualization allowing surgical skills to be easily mastered and shortening the learning curve. In our observation, the learning curve is highly influenced by the experience of the surgeon, degree of laparoscopic skills, the case volume the surgeon has, the number of cases the surgeon does in one day and the duration of time between procedures.

The aim of this study was to report the experience of two surgeons with different levels of training and experience with SSRC in a community teaching hospital. Study data comprise the initial experience of two surgeons from the same institution who treated 117 patients with varied gallbladder diseases. The demographics of our cohort were similar to the patient population of standard laparoscopic cholecystectomies: mainly women, obese, middle-aged and varied gallbladder diseases. No statistical demographic differences existed between the two surgeon series of patients. There were no complications reported in any of the procedures. None of our cases had unusual bleeding that required a blood transfusion. Furthermore, none of the cases in our series developed a bile leak, bile duct injury, pneumonia, pulmonary embolism, deep vein thrombosis or postoperative sepsis and there were no cases of retained bile duct stones or any that required reoperation (Table 2). Most of the previous studies on SSRC have reported no major complications [19]. Our perioperative outcomes are consistent with the accumulating knowledge with respect to the safety and feasibility of SSRC [20].

The conversion rate in our series was 4% for surgeon 1 compared to 20% for surgeon 2, which was statistically significant (*P* = 0.044). The reasons for conversion to laparoscopy in the series of patients of surgeon 1 included two cases of acute cholecystitis with significant inflammation, one case of the cystic duct tearing and one case where the right arm and the left arm were tangled in which they had to call the support team and did not receive help. The reason for conversion among surgeon 2’s patients included two cases of acute cholecystitis with significant inflammation and a case reporting the cannulas being too long, which made the dissection challenging.

To assess the learning curve differences in our series of patients for both surgeons, we compared the total operative times required to complete the procedure safely. The two series had the same inclusion and exclusion criteria.

The overall mean operative time of 49.3 ± 17.8 min remained acceptable and less than what has been reported in the literature, despite the differences that exists between the two surgeons. The total operative time significantly differed between the two surgeons (*P* < 0.001). Surgeon 1, who performed most of the procedures, showed statistically significant progress in their overall operative time due to advanced laparoscopic skills and case volume, in comparison to surgeon 2 who showed a progression in his total operative time, but his results were not significant due to the variability of the measures and the limited amount of data (Figs. 1 and 2). These results of operative time correlates significantly with the case volume, number of cases/day, and the time lapse between the procedures (Table 2).

Some studies [12, 21] have reported longer operative time in patients with acute cholecystitis compared to those without cholecystitis. Khambaty et al. [22] noted that the operative time tended to stabilize after the first 10 cases, finding a decrease from the initial time of 105.3 min to 74.4 min in the 10th case. In this series, the trends in the total operative time with the surgeon experience is demonstrated in Figs. 1 and 2. Surgeon 1 showed a continuous linear regression after 40 cases, while due to limited data it is non-conclusive for surgeon 2. Our hypothesis was that surgical skills would be more easily mastered and the learning curve would be shortened based on the laparoscopic skills of the surgeon, case volume and the number of cases the surgeon performed in a single day as well as the number of days between the procedures.

An expert surgeon in laparoscopic surgery might need a very short training period in SSRC to achieve an observed reduction in the operative time after the first 10 cases. Surgeons entering practice recently having basic laparoscopic skills will require a longer period of training and closed supervision before being able to achieve an acceptable learning curve.

It could easily be argued that the use of the robot for a cholecystectomy is unnecessary and too expensive. However, the robotic technique became widely popular and its use exceeded expectations. We believe that single-site robotic technology could be easily adapted to perform more complex intraabdominal procedures in a timely manner by more experienced surgeons with greater case volumes. Our data show some trends that do not reach statistical significance due to the relatively small sample size for surgeon 2, still this is a single-institutional data collection assessing the feasibility and safety of adopting a new technology, despite differences in surgeons’ experience.

However, this study has some limitations that need to be addressed. Since SSRC has been introduced recently, we are progressing in a learning mode and there will probably be some additional adjustments in terms of manipulations and optimization of all parts of the procedure, which could influence future operative times. We recommend that surgeons with basic laparoscopic skills and limited case volume have longer supervised SSRC training.

Other limiting factors included improper documentation of the docking time and console time. These factors further assist in the evaluation of the learning curve. Even though statistical significance was reached for some aspects, the findings represent an analysis of our initial experience with this procedure.

## Conclusion

It is recommended that SSRC can be performed with high standards of safety and efficacy, by general surgeons possessing both advanced and basic laparoscopic skills; however, it is advisable for surgeons of limited robotic skills and case volume to have more supervised single-site robotic cases until the learning curve is reached. SSRC provides surgeons with a system that possesses a gradual learning curve, atypical of the steep learning curve associated with learning new surgical procedures. Furthermore, we found that overall surgeon experience in laparoscopic cholecystectomy and amount of practice with SSRC procedures translated to decreased operation times. Future prospective, randomized trials are necessary to evaluate further benefits of this approach with respect to patient quality of life, satisfaction, cosmetics, and the advantage of significantly decreased postoperative pain, overall long- term umbilical sequelae and cost related issues in a community hospital setting.

## Abbreviations

SSRC: single-site robotic cholecystectomy
